# Bisphenol A and thyroid hormones: Bibliometric analysis of scientific publications

**DOI:** 10.1097/MD.0000000000023067

**Published:** 2020-11-06

**Authors:** Ning Yuan, Li Wang, Xiaomei Zhang, Wei Li

**Affiliations:** a Department of Endocrinology.; b Department of Pharmacy; c Department of General Surgery, Peking University International Hospital, Beijing, China.

**Keywords:** bibliometrics, Bisphenol A, thyroid hormones

## Abstract

Bisphenol A (BPA) is a well-known endocrine-disrupting chemical which can cause potential health risks and interfere with thyroid hormones through multiple avenues. This study aimed to evaluate the hotspots and emerging trends on BPA and thyroid hormones by using a bibliometric method.

Publications related on BPA and thyroid hormones were downloaded from Science Citation Index-Expanded database. Annual outputs, high yield journals, countries, institutions, authors and their cited times were summarized. In addition, keywords co-occurrence, burst references and citation networks were bibliometric analyzed.

From 2000 to 2019, 418 articles were published. Both of the *Environment International* and *Environmental Health Perspectives*, United States, Chinese Academy of Sciences and Antonia M. Calafat were the most recorded journals, countries, institutions and authors, respectively. The main research area was Toxicology. In addition of the retrieve term “bisphenol-a” and “thyroid-hormone”, “in-vitro”, “exposure” and “endocrine disruptors”, were the hotspot keywords and “triclosan”, “oxidative stress” and “united-states” were the most recent trends keywords. “Thyroid hormone action is disrupted by Bisphenol A as an antagonist” published on *The Journal of Clinical Endocrinology & Metabolism* by Kenji Moriyama in 2002 got both the highest burst score and citation score. Six groups were clustered and the mechanism of BPA's effect on thyroid hormones, and the exposure of BPA and potential risks in children and pregnant women were the two main large fields.

The number of publications in the field of BPA and thyroid hormones has increased tremendously since 2000. The research hotspot ranged from mechanism researches in animal models to epidemiological studies. “Thyroid hormone action is disrupted by bisphenol A as an antagonist” of Kenji Moriyama provided important building blocks in the field. The impact of BPA on thyroid hormones, especially pregnant women and children, was the latest research frontiers and might be the future direction of this filed in the following years.

## 1 Introduction

Bisphenol A (BPA, 4,4’-isopropylidenediphenol), which is used in industry to synthesize materials such as polycarbonate and epoxy resins, is found in everything from water bottles to medical devices of the package lining.^[1]^ BPA is a well- known endocrine-disrupting chemical (EDCs) which can cause potential health risks on human beings has become the focus of research. Thyroid hormones are essential for development, growth and metabolism, especially for neurodevelopment in children. BPA has estrogen-like effects and other contributing factors including damage to reproductive, endocrine and immune systems. Recently, mounting evidences indicate that BPA can interfere with thyroid hormone synthesis, transport, and metabolism through multiple avenues.^[2–5]^

Bibliometrics could measure scientific outputs through quality and quantity indicators, as well as map the scientific research through software tools from the bibliographic databases. Web of Science (WoS) Core Collection of Thomson Reuters is recognized as the most reliable database for bibliometric analysis based on its valuable and high-impact collection of data. Bibliometric software, as VOSviewer, CiteSpace and CitNetExplorer, could create visualizations based on network data.

Recently, the articles on BPA and thyroid hormones have published on academic journals and increased remarkably, while bibliometric analysis is rarely seen. Therefore, in order to evaluate the active countries, institutions, authors, journals, display the citation networks, and explore the research hotspots and future trends in this field, we performed a bibliometric analysis of articles on BPA and thyroid hormones.

## 2 Methods

Publications used in this study were indexed from the Science Citation Index-Expanded database. The retrieve set was: (“bisphenol A”) and (“thyroid hormones” or “calcitonin” or “dextrothyroxine” or “diiodotyrosine” or “monoiodotyrosine” or “thyroid” or “thyronines” or “diiodothyronines” or “triiodothyronine” or “reverse triiodothyronine” or “thyroxine”) as the topic, “article” as the document type while excluding Proceedings Paper or Book Chapter or Correction or Review or Meeting Abstract or Editorial Material, "1900–2019” as the timespan. The data of all eligible articles including full records and cited references, the records and citation reports of publication years, authors, affiliations, countries, source journals and research areas were downloaded from the database.

Based on the bibliographic data, VOSviewer (1.6.14 edition) was utilized to create the keywords co-occurrence, also known as the co-words overlay visualization; CiteSpace (5.5.R2 edition) was employed to calculate the references with the strongest citation bursts, and CitNetExplorer (1.0.0 edition) was used to provide the direct citation networks. The ethical approval was not necessary in this study.

## 3 Results

### 3.1 Annual outputs and annual times cited

**Figure 1:**
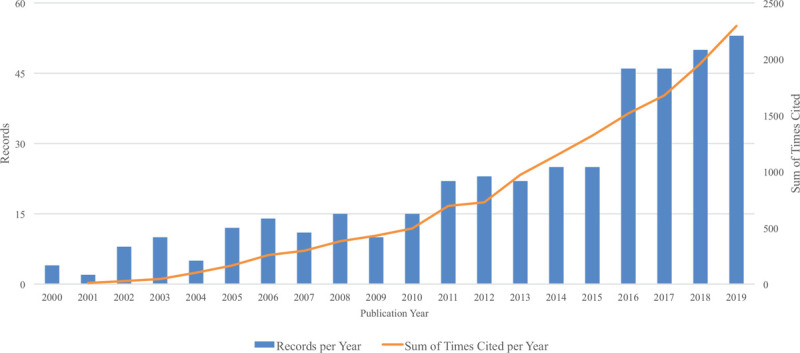
Annual records and annual sum of times cited on BPA and thyroid hormones research from 2000 to 2019. The column represents the annual publications, while the curve depicts the annual cited times.

The annual output is a production indicator for quantity analysis. Based on the search strategy, 418 academic articles about the BPA and thyroid hormones were indexed. Figure 1 showed the annual records and annual sum of times cited from 2000 to 2019. The first research was published in 2000 and the initial citation was started in 2001. Both the most publications (53 records) and citations (2296 cited times) year was 2019.

### 3.2 The productive journals, countries, institutions and authors

The citations including total citation (TC) and average citation (AC), the *h*-index from the WoS citation report, and the journal impact factor (IF), Journal Citation Report (JCR) category, rank and quartile (Q) are the quality indicators for bibliometric analysis.

#### 3.2.1 Journals

In total, 418 articles were published in 151 journals included in Science Citation Index. Table 1 demonstrated the 10 most productive journals (2 journals tied for 10th) with the record, *h*-index, TC, AC, IF of 2018, JCR category, rank and quartile. Both *Environment International* and *Environmental Health Perspectives* had the most record of 18 articles each, while the former had the highest IF of 2018 with 7.943, and the latter occupied the top *h*-index of 15 and TC of 1450. *Toxicological Sciences* owned the highest AC of 81.65. These journals were belonged to 6 JCR categories: ‘Environmental Sciences’, ‘Toxicology’, ‘Endocrinology & Metabolism’, ‘Marine & Freshwater Biology’, ‘Multidisciplinary Sciences’, and ‘Engineering, Environmental’, which were located Q1 and Q2 in each category except *General and Comparative Endocrinology* was in Q3.

**Table 1:** The 10 most productive journals on BPA and thyroid hormones research from 2000 to 2019.

							Journal Citation Report	
							
No.	Journal	Record	*h*-index	TC	AC	IF of 2018	Category	Rank (Quartile)
1	*Environment International*	18	8	444	24.67	7.943	Environmental Sciences	8/251(Q1)
2	*Environmental Health Perspectives*	18	15	1450	80.56	7.736	Environmental Sciences	2/93(Q1)
							Toxicology	10/251(Q1)
3	*Chemosphere*	17	10	328	19.29	5.108	Environmental Sciences	32/251(Q1)
4	*Toxicological Sciences*	17	13	1388	81.65	3.564	Toxicology	22/93(Q1)
5	*Endocrinology*	13	12	913	70.23	3.8	Endocrinology & Metabolism	45/145(Q2)
6	*Aquatic Toxicology*	12	11	503	41.92	3.794	Toxicology	17/93(Q1)
							Marine & Freshwater Biology	4/108(Q1)
7	*Environmental Research*	12	6	221	18.42	5.026	Environmental Sciences	33/251(Q1)
8	*Science of The Total Environment*	12	7	167	13.92	5.589	Environmental Sciences	27/251(Q1)
9	*PLOS ONE*	11	11	395	35.91	2.776	Multidisciplinary Sciences	24/69(Q2)
10	*Environmental Science Technology*	10	9	530	53	7.149	Environmental Sciences	14/251(Q1)
							Engineering, Environmental	5/52(Q1)
10’	*General and Comparative Endocrinology*	10	8	370	37	2.445	Endocrinology & Metabolism	95/145(Q3)

#### 3.2.2 Countries

In total, 418 articles were published from 48 countries. Table 2 displayed the top 10 productive countries with record, proportion, *h*-index, TC and AC. United States ranked the top with record of 124 articles, *h*-index of 40 and TC of 5372. The Netherlands had the highest AC of 101.83 even though it claimed 18 records.

**Table 2:** The top 10 productive countries on BPA and thyroid hormones research from 2000 to 2019.

Rank	Country	Record	Proportion (%)	H-index	TC^∗^	AC^†^
1	USA	124	29.74	40	5372	43.32
2	Peoples Republic of China	83	19.90	22	1575	18.98
3	Japan	59	14.15	28	3190	54.07
4	Canada	31	7.43	16	758	24.45
5	France	23	5.52	13	685	29.78
6	South Korea	23	5.52	13	703	30.57
7	Germany	22	5.28	17	1166	53.00
8	Italy	19	4.56	13	704	37.05
9	the Netherlands	18	4.32	16	1833	101.83
10	England	13	3.12	9	859	66.08

#### 3.2.3 Institutions

In total, 418 published articles came from 689 institutions. Table 3 showed the top 10 productive institutions with record, proportion, *h*-index, TC and AC. Chinese Academy of Sciences (Chinese Acad Sci) with 18 records located first on the list. Vrije Universiteit Amsterdam obtained the highest TC of 1441, AC of 131. Centers for Disease Control & Prevention United States (Ctr Dis Control Prevent) had the highest *h*-index of 11 as well as the other four institutions, Harvard University, Nanjing Medical University, University of California System, and University of Michigan.

**Table 3:** The top 10 productive institutions on BPA and thyroid hormones research from 2000 to 2019.

Rank	Institution	Record	Proportion (%)	H-index	TC^∗^	AC^†^
1	Chinese Academy of Sciences	18	4.32	10	283	15.72
2	Centers for Disease Control Prevention USA	16	3.84	11	1012	63.25
3	Research Center for Eco Environmental Sciences	16	3.84	9	259	16.19
4	Harvard University	14	3.36	11	794	56.71
5	Nanjing Medical University	14	3.36	11	385	27.50
6	University of California System	14	3.36	11	560	40.00
7	University of Michigan	14	3.36	11	816	58.29
8	University of Cincinnati	13	3.12	8	464	35.69
9	Brown University	11	2.64	6	229	20.82
10	Vrije Universiteit Amsterdam	11	2.64	10	1441	131.00

#### 3.2.4 Authors

In total, 418 published articles belonged to 2043 authors. Table 4 showed the top 5 productive authors with record, proportion, *h*-index, TC and AC. Antonia M. Calafat ranked first based on the most record of 16 papers, the highest *h*-index of 11 and TC of 1012. John D. Meeker also had the *h*-index of 11 with 13 papers. Robert Thomas Zoeller had the highest AC of 77.4 based on the 10 published articles.

**Table 4:** The top 5 productive authors on BPA and thyroid hormones research from 2000 to 2019.

Rank	Author	Record	Proportion (%)	H-index	TC^∗^	AC^†^
1	Antonia M. Calafat	16	3.83	11	1012	63.25
2	John D. Meeker	13	3.11	11	815	62.69
3	Li Yuan-yuan	11	2.63	6	92	8.36
4	Joseph M. Braun	10	2.39	5	107	10.70
5	Zoeller Robert Thomas	10	2.39	7	774	77.40

### 3.3 Research areas

**Figure 2:**
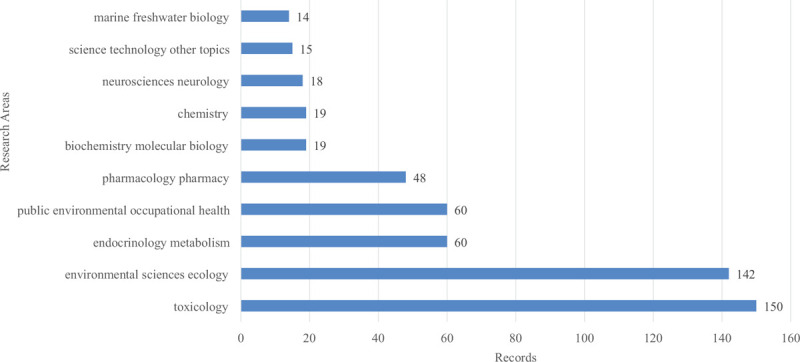
The top 10 research areas on BPA and thyroid hormones research from 2000 to 2019. The column illustrates the article record in each research area.

In total, 418 published articles were distributed in 44 research areas. One publication could belong to more than one research areas. Figure 2 shows the top 10 research areas on this topic from 2000 to 2019. Followed by Endocrinology Metabolism and public environmental occupational health each of 60 records, Toxicology and Environmental Sciences Ecology occupied the top 2 spots with 150 and 142 records, respectively.

### 3.4 Keywords co-occurrence

**Figure 3:**
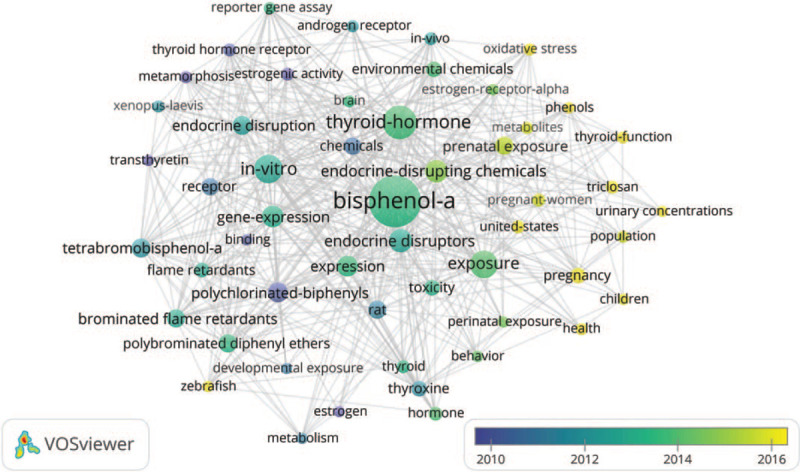
Keywords co-occurrence overlay visualization on BPA and thyroid hormones research from 2000 to 2019. Circles represent keywords, the size is directly proportional to the occurred times, and the color is associated with the average publication year. Lines mean the co-occurrence times.

Keywords which are composed of the “author keywords” provided by authors and the “keywords plus” supplied by journals in WoS appear together in a publication could be related in a network. A total of 2471 words were marked in the 418 articles. Singular and plural, full name and abbreviation words were substituted to conduct data cleaning. Figure 3 provided the overlay visualization of the top 52 co-word which were defined as occurred more than 15 times in all articles. The higher frequency (freq) of a keyword, the larger is the label and the circle. Lines between keywords represented links and relatedness. The color of a keyword is determined by the average publication year, where colors range from blue (farther year) to green to yellow (recent year) by default. “bisphenol-a” (freq = 304), “thyroid-hormone” (freq = 131), “in-vitro”(freq = 94), “exposure” (freq = 86) and “endocrine disruptors” (freq = 64) were the top 5 high frequency keywords, while “triclosan”, “oxidative stress”, and “united-states” in 2017, “pregnancy”, “thyroid-function”, “phenols”, “health”, “urinary concentrations”, and “zebrafish” in 2016 had the most recent average publication year.

### 3.5 Burst references

The appearance of a topic is signaled by “citation burst” which indicated the rapidly occurring in a network data in certain periods.^[6]^Table 5 demonstrates the top 20 references with the strongest citation bursts. The highest burst strength of 9.9836 belonged to Kenji Moriyama, 2002.^[7]^ The earliest burst begin year was 2002 belonged to Ilonka A. T. Meerts, 2000.^[8]^ The longest burst timespan of 7 years belonged to Robert Thomas Zoeller, 2005 and Shigeyuki Kitamura, 2005.^[9,10]^ The latest burst articles were belonged to Wang Tiange, 2013.^[11]^

**Table 5:** The top 20 References with the Strongest Citation Bursts.

References^∗^	Strength	Begin	End	2000–2019
Meerts, 2000	6.069	2002	2008	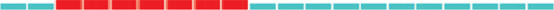
Cheek, 1999	4.448	2003	2006	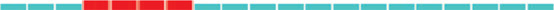
Brucker-Davis, 1998	3.4179	2003	2006	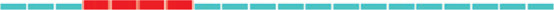
Moriyama, 2002	9.9836	2005	2010	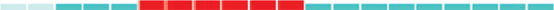
Iwamuro, 2003	3.0953	2005	2011	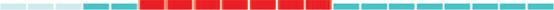
Schonfelder, 2002	3.0876	2005	2010	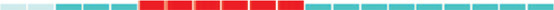
Seiwa, 2004	4.2164	2005	2011	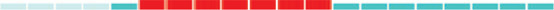
Zoeller, 2005	3.1467	2006	2013	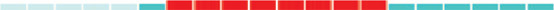
Kitamura, 2005	4.31	2006	2012	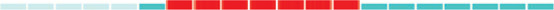
Kitamura, 2005	9.254	2006	2013	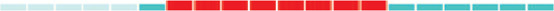
Hamers, 2006	4.1582	2007	2013	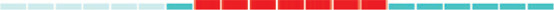
Calafat, 2008	5.0458	2009	2015	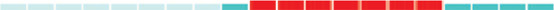
Vandenberg, 2007	5.7728	2009	2015	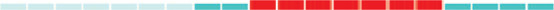
Boas, 2006	3.8606	2009	2013	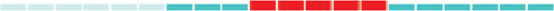
Diamanti-Kandarakis, 2009	4.2662	2010	2016	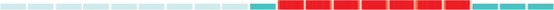
Heimeier, 2009	3.635	2011	2015	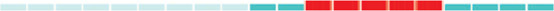
Lang, 2008	3.1371	2011	2015	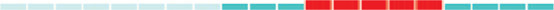
Vandenberg, 2009	4.5635	2011	2015	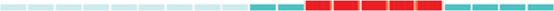
Meeker, 2011	4.1434	2015	2017	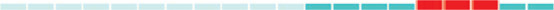
Wang, 2013	3.4378	2015	2017	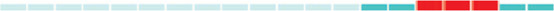

### 3.6 Citation networks

**Figure 4:**
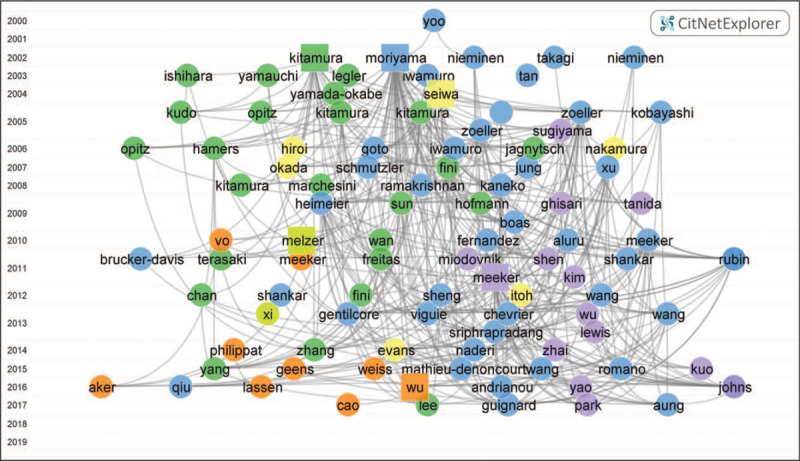
The citation network on BPA and thyroid hormones research from 2000 to 2019. Each circle or square delineates a publication which is labeled with the last name of the first author. Lines mean the citation relation. Colors cluster the 6 groups, in which square represents the article with the highest citation score.

Based on the algorithmic historiography designed by Eugene Garfield,^[12]^ CitNetExplorer was designed to delineate the development of a research field over time and cluster the research sub-areas. Figure 4 illustrates the citation network of top 100 publications on BPA and thyroid hormones. Six main groups were clustered based on 1489 citation links as the minimum group size was set to include 10 publications. Each circle labels a publication with the last name of the first author, while square represents the highest citation score article in each group in the visualization. The largest group 1 (blue) contains 142 records while “Thyroid hormone action is disrupted by bisphenol A as an antagonist” of Kenji Moriyama with the highest citation score of 118.^[7]^ Group 2 (green) contains 98 records while “Thyroid hormonal activity of the flame retardants tetrabromobisphenol A and tetrachlorobisphenol A” of Shigeyuki Kitamura with the highest citation score of 41.^[13]^ Group 3 (purple) contains 44 records while “Relationship between urinary phthalate and bisphenol A concentrations and serum thyroid measures in U.S. adults and adolescents from the national health and nutrition examination survey (NHANES) 2007–2008” of John D. Meeker with the highest citation score of 43.^[4]^ Group 4 (orange) contains 40 records while “Effect of triclosan, triclocarban, 2,2 ’,4 ’,-tetrabromodiphenyl ether, and bisphenol a on the iodide uptake, thyroid peroxidase activity, and expression of genes involved in thyroid hormone synthesis” of Wu Yuanfeng with the highest citation score of 14.^[14]^ The rest 2 groups (yellow and lime) contain 26 an 11 publications, while “Bisphenol A exerts thyroid-hormone-like effects on mouse oligodendrocyte precursor cells” of Chika Seiwa and “Association between serum perfluorooctanoic Acid (PFOA) and thyroid disease in the US national health and nutrition examination survey” of David Melzer with citation score of 20 and 5, respectively.^[15,16]^

## 4 Discussion

Figure 1 showed that the number of publications in the field of BPA and thyroid hormones had increased tremendously over a quarter century. Since 2008, the annual publication volume had gradually increased over 15 for the first time. However, papers published between 2016 and 2019 account for half of the total published since 2000. The published articles were supported by research funds since 2005. The increasing of the published article numbers was associated with the increasing of research funds. National Natural Science Foundation of China, National Institutes of Health United States, Department of Health Human Services contributed the most investments. Along with the effects of BPA on thyroid hormones were concerned, some scholars started to research in this field.

The total number of articles published by the top 10 magazines (2 journals tied for 10th) ranged from 10 to 18 articles. They all had an IF of 2018 > 2.0, while 6 out of the 11 active journals had an IF > 5 (2018). All the six journals from the Environmental Sciences, all the three journals from Toxicology, and each one journal from Marine & Freshwater Biology and Engineering Environmental, were all rank in Q1 of their own categories. They were both high production and high-quality journals on this topic. Therefore, the researches of BPA and thyroid hormones were a relatively new research area and the most cutting-edge studies. While the only two journals from the category of Endocrinology & Metabolism were rank in Q2 and Q3, respectively. The results reminded the endocrinologist to pay more attention to the effect of BPA on thyroid hormones. The analysis of published journals was helpful for scholars to select journals when reading literature or submitting articles.

The total number, frequency of citations and *h*-index represent a country's quality of publications and academic impact. The United States contributed the most to the global publications in terms of total number of publications, citation frequency and *h*-index. Three of the top 10 countries came from Asia and the others came from Europe and America. China was the only developing country, which ranked 2^nd^ in the total number of publications, but 3rd in *h*-index and 4th in citation frequency. Japan ranked 3^rd^ in the number of total publications, however, was higher than China in citation frequency and the h-index. Studies of BPA and thyroid hormones began in the United States and Japan since 2000, while the Chinese studies only began in 2008. In recent years, with the development of China's economy and the expansion of research funds in this field, China's output was increasing gradually.

Among the high yield organizations, Chinese Acad Sci, founded in 1949, was the highest academic institution of natural science and the comprehensive research and development center of natural science and high technology in China. Chinese Acad Sci had published 18 articles about BPA and thyroid hormones since the first one about thyroxine transport disruption by BPA published in 2011.^[17]^ Most articles were the basic studies about mechanism of BPA and thyroid hormones and published in toxicology and environmental sciences ecology areas.^[18,19]^ Ctr Dis Control Prevent ranked 2nd in the total number of publications, but 1st in *h*-index. Most articles were epidemiological research about the effects of BPA on human and thyroid hormones. The pregnancy women and children were the special community which Ctr Dis Control focused on.^[20]^ Nanjing Med University had published 14 articles since 2008. The studies included not only basic research about animal experiment and gene assays, but also some epidemiological research about occupational exposure and obesity.^[21,22]^ Vrije Universiteit Amsterdam ranked the 10th in the total number of publications, but the 1st in TC and AC. The topic of the institution was mainly involved basic research about endocrine-disrupting chemicals.

Among the top 5 authors, Antonia M. Calafat came from Ctr Dis Control Prevent, United States, contributed the most publications. Those articles were about the effect of BPA on reproduction and thyroid hormones.^[23]^ The physiological and psychosocial effects of BPA in children were getting inordinate amounts of this scholar.^[24,25]^ John D. Meeker came from University of Michigan, United States, ranked 2nd in the number of articles published. Most articles involved the relationship between BPA and thyroid hormones in pregnant women, children and adolescents.^[4,26]^ Both of the former two authors had the highest *h*-index of 11. Li Yuanyuan came from Chinese Acad Sci and ranked 3nd in the number of articles published. Robert Thomas Zoeller came from University of Michigan and found that the expression of the TH-responsive gene RC3/neurogranin was significantly up-regulated by BPA in the dentate gyrus.^[27]^ This article has been cited more than 300 times and had the highest citation frequency.

The purpose of analytical research areas was to explore which institutions and experts concerned with this research. The articles about BPA and thyroid hormones were mainly published in toxicology and environmental sciences ecology areas. Endocrinology metabolism ranked 3rd in the research areas. Therefore, scholars concerned about the toxicology and environmental effects of BPA, and its endocrine effects.

Citation burst was defined as a feature rising sharply in frequency in certain periods in a dataset.^[28]^ There were reviews and articles among top 20 references with the strongest citation bursts. Although the literature type included in this study were articles, some reviews were cited in these articles. Consequently, reviews with high citations were classed as top 20 references with the strongest scores. An Endocrine Society scientific statement about EDCs in 2009^[29]^ showed that EDCs which were substances in our environment, food, and consumer products, threated human's health and affected the thyroid hormones. The statement increased understanding of effects of EDCs and advocated people to concern the health impact of EDCs. Therefore, this statement was repeatedly cited by other articles between 2010 and 2016. A review of the multiple effects of BPA was published in 2011 and had been cited more than 200 times.^[30]^ This paper reviewed the adverse reactions of BPA and discussed the possible mechanism of action. A separate review about low-dose effects and non-monotonic dose responses of hormones and EDCs was cited more than 500 times by other articles.^[31]^ In order to protect human health, fundamental changes in chemical testing and safety determination were requisite. However, the effects of low doses cloud not be forecasted by the effects observed at high doses when non-monotonic dose-response curves used. A review published in 2012 showed that phthalates, BPA, brominated flame retardants and perfluorinated chemicals might exert thyroid effects through a variety of mechanisms of action.^[32]^ The latest review about BPA and human health was cited more than 200 times by the following articles.^[33]^ This review outlined the previous 91 studies and showed the associations between BPA exposure and adverse perinatal, childhood, and adult health outcomes. Reviews which contributed to summarize previous studies, put forward new ideas and predicate research trends, were often quoted repeatedly in the article.

Among reviews with the strongest citation bursts, an Endocrine Society scientific statement began to advocate people to concern the health impact of EDCs. The study on the EDCs and health had since increased rapidly. Recently, researchers had focused on the effects of BPA on thyroid hormones, pregnancy and child health.

Studies of BPA and thyroid hormones mainly covered the animal models, human clinical observations, and epidemiological studies. In animal studies, the mechanism of BPA's effect on thyroid hormones had been investigated from different perspectives including the ability to antagonize thyroid hormone gene regulation and its role as a thyroid hormone receptor antagonist.^[7,34,35]^ Moriyama et al found that BPA can cause gene suppression by replacing T (3) from TR and recruiting a transcriptional repressor.^[7]^ The article was the first report that BPA can antagonize T (3) action at the transcriptional level and marked a milestone in the field of BPA and thyroid hormones. Urine concentrations of total BPA varied by race/ethnicity, age, sex, and household income. In 2008, a research first reported the concentration data for urinary BPA in United States population and was cited more than 500 times by the following articles.^[36]^ Studies in China and the United States supported previous studies of associations between BPA exposure and thyroid hormones changes in animal models.^[4,11]^ Additionally, prenatal exposure to BPA may perturb thyroid function, including reduce total T4 in pregnant women and decrease TSH in neonates.^[20,37]^ Since 1999, effects of BPA on thyroid hormones had caused vast attention. The studies ranged from mechanism researches in animal models to epidemiological studies in pregnant women and children.

Statistics and analysis of the co-occurrence words in scientific publications can provide an intuitive picture of the veritable content of our study. As shown in Figure 3, the keywords changed over time. From 2000 to 2010, the keywords “thyroid hormone receptor” “rat” and “estrogenic activity” suggested that thyroid hormone receptor was involved in the pathogenesis of animal models.^[38]^ From 2010 to 2014, “gene-expression” “endocrine disruptors” and “in vitro” became the keywords. The gene expression became a new hot research point in the pathogenesis of the BPA and thyroid hormones.^[39]^ Since 2016, research hotspots of BPA and thyroid hormones switched to “prenatal exposure” “pregnant women” and “children”.^[40,41]^ The trend of BPA and thyroid hormones with the change in time was studied to preface some new orientations in this field.

CitNetExplorer was beneficial to further research and cluster analysis of the literatures. As shown in Figure 4, the top 100 publications concerning BPA and thyroid hormones and six main groups cluster were presented by CitNetExplorer in this study. The largest group (blue) contained 142 records which were published from 2000 to 2017. This group mainly included the interference of BPA on thyroid hormones,^[7]^ exposure of BPA and potential risks in infants, children and pregnant women,^[42]^ and the association of BPA with thyroid disease.^[43]^ The group 2 (green) mainly referred the mechanism of BPA's effect on thyroid hormones.^[35]^ The group 3 (purple) contained 44 records and the first article was published in 2005. This group showed the relationship between the maternal BPA exposure and thyroid hormones in pregnant women and their offspring. The first article of the group 4 (orange) was published in 2010. The core discussion of this group was about the effect of BPA on pubertal development, reproduction, and child behavior and neurodevelopment.^[24,44]^ The group 5 (yellow) included 26 publications and involved the effect of BPA on the neuroendocrine system. The first article of group 6 (lime) published in 2010. This group only contained 11 publications and exhibited the influence of nonylphenol exposure on thyroid tissue structure and thyroid function.^[16]^

This study was focused on BPA and thyroid hormone, however, there were other endocrine disrupting chemicals/molecules, such as phytosterols, epigallocatechin-3-gallate (ECGC), anacardic acid, zinc, berberine, etc. The phytosterol-containing treatments, which was shown to increase the levels of total thyroxine, total triiodothyronine, and free triiodothyronine, increased activity of thyroid glands.^[45]^ EGCG can be used to treat thyroid cancer through suppressing the EGFR/RAS/RAF/MEK/ERK signaling pathway.^[46]^

There are limitations in this study. The primary results are grounded on statistical analysis of academic publications in the WoS, which is a comprehensive database and able to provide information of journal sources, country, institution, and author, however some articles in BPA and thyroid hormones could be missed in other scientific journals. The WoS might also bring some bias by over-representing journals in English.

## 5 Conclusion

The number of publications in the field of BPA and thyroid hormones has increased tremendously since 2000. The United States contributed the most to the global publications in terms of total number of publications, citation frequency and *h*-index. Vrije Universiteit Amsterdam and Antonia M. Calafat were the most influential institution and author. *Environment International* and *Environmental Health Perspectives* published the highest number of articles in this field. The research hotspot ranged from mechanism researches in animal models to epidemiological studies. “Thyroid hormone action is disrupted by bisphenol A as an antagonist” of Kenji Moriyama provided important building blocks in the field. The impact of BPA on thyroid hormones, especially pregnant women and children, was the latest research frontiers and might be the future direction of this filed in the following years.

## Acknowledgments

At the point of finishing this paper, I would like to show my sincere thanks to all those who helped me during the course of writing this paper.

## Author contributions

**Conceptualization:** Ning Yuan, Li Wang.

**Data curation:** Ning Yuan, Li Wang, Xiaomei Zhang.

**Formal analysis:** Ning Yuan, Li Wang.

**Funding acquisition:** Ning Yuan, Xiaomei Zhang.

**Investigation:** Ning Yuan, Li Wang.

**Methodology:** Ning Yuan, Li Wang, Xiaomei Zhang.

**Project administration:** Wei Li.

**Resources:** Ning Yuan, Li Wang.

**Software:** Li Wang.

**Supervision:** Wei Li.

**Validation:** Ning Yuan, Li Wang.

**Visualization:** Ning Yuan, Li Wang.

**Writing – original draft:** Ning Yuan, Li Wang.
